# A Magnesium Carbonate Hydroxide Nanofiber/Poly(Vinylidene Fluoride) Composite Membrane for High-Rate and High-Safety Lithium-Ion Batteries

**DOI:** 10.3390/polym15204120

**Published:** 2023-10-17

**Authors:** Lin Luo, Kang Ma, Xin Song, Yuling Zhao, Jie Tang, Zongmin Zheng, Jianmin Zhang

**Affiliations:** 1 College of Mechanical and Electrical Engineering, National Engineering Research Center for Intelligent Electrical Vehicle Power System (Qingdao), Qingdao University, Qingdao 266071, China; llendure@163.com (L.L.); m15169580496@163.com (K.M.); songxin0625@163.com (X.S.); 2 State Key Laboratory of Bio Fibers and Eco Textiles, Qingdao University, No. 308 Ningxia Road, Qingdao 266071, China; zhaoyl_310@163.com; 3 National Institute for Materials Science, Tsukuba 305–0047, Japan; tang.jie@nims.go.jp

**Keywords:** magnesium carbonate hydroxide nanofiber, composite membrane, lithium-ion batteries, high rate, high safety

## Abstract

Simultaneously high-rate and high-safety lithium-ion batteries (LIBs) have long been the research focus in both academia and industry. In this study, a multifunctional composite membrane fabricated by incorporating poly(vinylidene fluoride) (PVDF) with magnesium carbonate hydroxide (MCH) nanofibers was reported for the first time. Compared to commercial polypropylene (PP) membranes and neat PVDF membranes, the composite membrane exhibits various excellent properties, including higher porosity (85.9%) and electrolyte wettability (539.8%), better ionic conductivity (1.4 mS·cm^−1^), and lower interfacial resistance (93.3 Ω). It can remain dimensionally stable up to 180 °C, preventing LIBs from fast internal short-circuiting at the beginning of a thermal runaway situation. When a coin cell assembled with this composite membrane was tested at a high temperature (100 °C), it showed superior charge–discharge performance across 100 cycles. Furthermore, this composite membrane demonstrated greatly improved flame retardancy compared with PP and PVDF membranes. We anticipate that this multifunctional membrane will be a promising separator candidate for next-generation LIBs and other energy storage devices, in order to meet rate and safety requirements.

## 1. Introduction

The development of energy storage devices has been on a fast track due to the rapid growth of renewable and clean energy sources. Lithium-ion batteries (LIBs) are the most well-established and studied energy storage devices, thanks to their favorable characteristics including good cycle performance, high working voltage, high energy density, power density, and environmental friendliness [1,2,3,4]. However, concerns about the safety of electric vehicles still persist due to potential hazards such as short circuits and fires caused in LIBs [5]. As a result, there is a pressing need to enhance both energy/power density and safety in battery technology.

The technical key constituents of LIBs primarily include positive electrode materials, negative electrode materials, separators, electrolytes, and assembly technology [6,7]. Among these, the separator is a pivotal component that plays a crucial role in the performance of the battery. It serves three key functions: (1) preventing direct contact between the positive and negative electrodes, which can cause short circuits; (2) facilitating the transportation of lithium ions between the positive and negative electrodes; and (3) storing electrolytes and ensuring the free passage of lithium ions through the electrolyte in order to complete the electrode reaction [8,9,10,11]. Polyolefin microporous membranes, specifically polyethylene (PE), polypropylene (PP), and sandwiched PP/PE/PP membranes, have emerged as common separator materials in LIBs due to their low cost, exceptional tensile strength, and strong chemical and electrochemical stability [12]. However, these membranes possess certain limitations, including poor electrolyte wettability and low thermal stability. Poor electrolyte wettability may cause various problems such as capacity deterioration, poor rate performance, and even encouraging lithium dendrites. Low thermal stability may lead to severe shrinkage at high temperatures, which increases the risks of thermal runaway situations [13,14]. To mitigate these risks, researchers have made numerous attempts to develop an ultimate solution. Robust polymers have emerged as promising candidates for LIBs separators, with a notable focus on poly(vinylidene fluoride) (PVDF) [15,16,17,18,19], polyimide (PI) [20,21,22], and poly(acrylonitrile) (PAN) [23,24]. PVDF, in particular, possesses exceptional electrochemical and thermal stability. In addition, the β crystalline phase of semicrystalline PVDF (TTTT molecular configuration) displays a high dielectric constant and polarity, which promotes efficient dissociation and transportation of lithium ions within the electrolyte, thereby leading to higher ionic conductivity [25]. Despite its favorable properties, pure PVDF membranes still cannot fulfill the thermally safe requirements for contemporary commercial applications. Researchers have taken advantage of adding fillers to enhance their performance. A promising approach involves utilizing the intrinsic characteristics of nanoparticles or nanosheets, including SiO_2_ [26,27], Al_2_O_3_ [28,29], Mg(OH)_2_ [30,31], Ni(OH)_2_ [32,33], and others, to bolster the electrolyte wettability and thermal stability of membranes. However, nanoparticles/nanosheets tend to aggregate, resulting in pore blockages and uneven membranes thickness. Moreover, in cases of various abuse conditions resulting in combustion, commercial membranes are lacking in flame-retardant solutions.

While attempts to enhance the safety of LIBs have yielded some success, they tend to focus on singular concerns. Here, we propose an innovative way of addressing multiple functions simultaneously by incorporating magnesium carbonate hydroxide (MCH) nanofibers into the PVDF matrix and utilizing a non-solvent-induced phase separation (NIPS) technique to produce a MCH/PVDF composite membrane. Our research delves into the comprehensive effects of different MCH nanofiber compositions on morphology, electrolyte affinity, lithium ion conductivity, lithium dendrites’ suppression, the thermal stability of composite membrane, and the electrochemical performance of the assembled LiFePO_4_/membrane/Li half-cells.

## 2. Experimental

### 2.1. Materials

PVDF (Solef 6010) was purchased from Solvay Co. (Tavaux, France). N,N-dimethylformamide (DMF, analytical grade), magnesium acetate (Mg(CH_3_COO)_2_·4H_2_O), and carbamide (CH_4_N_2_O) were brought from Sinopharm Chemical Reagent (Shanghai, China) Co., Ltd. N-methyl-2-pyrrolidone (NMP, analytical grade) and 1 mol/L LiPF_6_ (in EC:DEC = 1:1 *v*/*v*) were brought from Suzhou Duoduo Chemical Technology (Suzhou, China) Co., Ltd. All chemicals were used without further purification and modification.

### 2.2. Synthesis of Magnesium Carbonate Hydroxide Nanofibers

A solution was prepared by mixing 500 mg Mg(CH_3_COO)_2_·4H_2_O and 2.4 g CH_4_N_2_O with 100 mL of water and stirring for 5 min at room temperature (25 °C). Next, the mixture solution was heated in a reactor at 180 °C for 2 h, then cooled to room temperature. The pure magnesium carbonate hydroxide (MCH) nanofibers were then obtained by filtering and drying the products at 110 °C for 12 h.

### 2.3. Preparation of Composite Membranes

**Figure 1:**
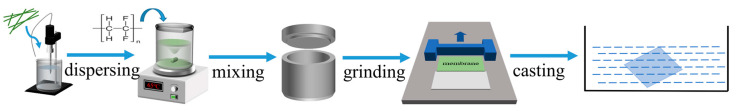
Schematic of the fabrication process of MCH/PVDF composite membranes.

This study used the non-solvent-induced phase separation (NIPS) method to create composite membranes, as shown in Figure 1. MCH nanofibers were mixed into DMF and sonicated for 20 min. PVDF was then added to the suspension and dissolved under magnetic stirring at 50 °C for 3 h to make a blended slurry. The blended slurry was evenly mixed by ball-milling. After degassing at 30 °C for 2 h, the slurry was cast onto a glass plate using a doctor blade. The plate was immersed in deionized water, and the solvent (DMF) in the solution underwent an exchange with the non-solvent (water), resulting in liquid–liquid phase separation. This led to a phase transition of the original stable solution, hence the polymer thin layer solidified into a porous membrane [34]. MCH/PVDF composite membranes were obtained after drying in a vacuum oven at 60 °C for 12 h. Weight ratios of MCH nanofibers of 10 wt%, 30 wt%, and 50 wt%, respectively, were used to investigate the effect of MCH nanofiber content on various properties of composite membranes. Samples with different amounts of MCH were sequentially denoted PM-10, PM-30, and PM-50, respectively. The samples used for comparison were a commercial PP membrane (Celgard 2500, Charlotte, NC, USA) and a neat PVDF membrane.

### 2.4. Characterizations

X-ray diffraction (XRD, Ultima IV, Tokyo, Japan) was carried out to analyze the crystalline structure of nanofibers at diffraction angles, within the range of 5−80°. The morphologies and microstructures of the composite membranes were analyzed with a scanning electron microscope (SEM, JSM-6390LV, Tokyo, Japan) at an accelerating voltage of 15 kV, along with energy-dispersive spectroscopy (EDS).

Contact angles were measured using the sessile drop method with 5 μL electrolyte on a contact angle meter (Theta Flex, Gothenburg, Sweden). Electrolyte uptake was determined by weighing the membranes before and after 2 h of soaking, and calculating the uptake ratio using Equation (1):

(1) $$\eta =\frac{M_{2}-M_{1}}{M_{1}}\times 100\%$$

where M_1_ and M_2_ are the weights of the membrane before and after soaking, respectively. Membrane porosity was determined using the n-butanol soaking method, and calculated by following Equation (2).

(2) $$P=\frac{M_{2}-M_{1}}{\rho \times A\times D}\times 100\%$$

where ρ is the density of the n-butanol solvent. M_1_ and M_2_ are the weights of the membrane before and after soaking for 2 h, and A and D are the area and thickness of the membrane, respectively.

The thermal decomposition behavior of the membranes was analyzed using thermogravimetry (TG, STA8000, Shelton, CT, USA) under a nitrogen atmosphere, heating from room temperature to 800 °C at a rate of 10 °C·min^−1^. Thermal shrinkage behavior was determined by heating the membranes at various temperatures for 2 h, and the resulting shrinkage ratio was calculated using Equation (3).

(3) $$TSR=\frac{S_{0}-S_{1}}{S_{0}}\times 100\%$$

where S_0_ and S_1_ are the areas of membranes before and after heat treatment, respectively.

The limiting oxygen index (LOI) value of various membranes was measured using the oxygen index test instrument (FTT0077, West Sussex, UK), according to the GB/T 2406.2 standard [35].

### 2.5. Electrochemical Measurements

The cathode materials were prepared by mixing LiFePO_4_, PVDF, and Super P powders in an 8:1:1 weight ratio with NMP solvent. The electrochemical performance of the LiFePO_4_/membrane/Li half-cells with various membranes was evaluated using CR2032 coin cells. The electrolyte used in this study was 1 mol/L LiPF_6_ (in EC: DEC = 1:1 *v*/*v*). Cells were assembled in an argon-filled glove box with low moisture (<0.1 ppm) and oxygen (<0.1 ppm). Charge/discharge cycling performance and C-rate capability were tested using a Neware Battery Testing System (BTS-4000, Shenzhen, China) over a voltage range of 2.5–4.2 V at 25 °C and 100 °C, respectively.

To assess the electrochemical stability of the membranes, linear sweep voltammetry (LSV) was performed. Stainless steel working electrodes were sandwiched with the membranes and lithium metal counter electrodes before testing at a scan rate of 0.1 mV·s^−1^ at 25 °C.

The electrochemical impedance spectroscopy (EIS) method was used to measure the bulk resistance (R_b_) of the SS/membrane/SS cell. The measurements were taken at a voltage amplitude of 5 mV and in a frequency range of 0.1–500 kHz at 25 °C. Using Equation (4), we calculated the ionic conductivity (σ).

(4) $$\sigma =\frac{D}{R_{b}\times A}$$

where D is the thickness of the membrane, A is the surface area, and R_b_ is the bulk resistance, respectively. 

The lithium ion transference number was evaluated via chronoamperometry and EIS. Specifically, the resistance of the Li/membrane/Li cell before and after polarization was tested via AC impedance. The polarization was completed for 2500 s under a voltage of 10 mV, and it was calculated using the following Equation (5):

(5) $$t_{{\mathrm{Li}}^{+}}=\frac{I_{S}(\Delta V-I_{0}R_{0})}{I_{0}(\Delta V-I_{S}R_{S})}$$

where ΔV is 10 mv, and I_0_, I_S_, R_0_, and R_s_ represent the initial current, the steady-state current, the initial resistance, and the steady-state resistance, respectively.

The charge transfer resistance (R_ct_) of the Li/membrane/Li cell at different temperatures (30, 35, 40, 45, and 50 °C) was determined using EIS. The measurements were carried out at different temperatures with a voltage amplitude of 5 mV and a frequency range of 0.1 Hz–500 kHz. We used the Arrhenius equation (Equations (6) and (7)) to calculate the activation energy required for lithium ion transport at the interfaces of the electrode and electrolyte-absorbing membrane.

(6) 1Rct=Ae−EaRT

(7) $$\ln\left(\frac{1}{R_{\mathrm{ct}}}\right)=\ln A-\;\frac{E_{a}}{R}\times \frac{1}{T}$$

where A is a pre-exponential factor, R_ct_ represents the interfacial impedance (Ω), T is the temperature (K), and R is 8.314 J K^−1^ mol^−1^; E_a_ is the activation energy for lithium ion transport.

## 3. Results and Discussion

### 3.1. Characterization of Magnesium Carbonate Hydroxide (MCH) Nanofiber

**Figure 2:**
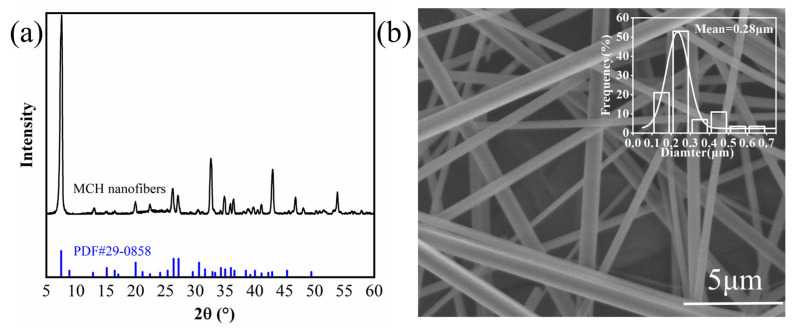
(**a**) XRD profile and (**b**) SEM image of MCH nanofibers.

The size, morphology, and crystalline structure of the as-prepared MCH nanofibers were analyzed using SEM and XRD. The XRD profile in Figure 2a reveals that all reflection peaks correspond to 4MgCO_3_·Mg(OH)_2_·5H_2_O (JCPDS card No. 29-0858). Figure 2b shows that the MCH nanofibers have fibrous structures with diameters ranging from 150 to 450 nm. The formation of these MCH nanofibers can be expressed through the following major chemical reactions:

$$\mathrm{CO}{(NH_{2})}_{2}+H_{2}O\to CO_{2}+NH_{4}^{+}+OH^{-}\;{\mathrm{Mg}}^{2+}+CO_{2}+OH^{-}\to 4\mathrm{MgC}O_{3}\cdot \mathrm{Mg}{\left(\mathrm{OH}\right)}_{2}\cdot 5H_{2}O$$

### 3.2. Morphology of Composite Membranes 

**Figure 3:**
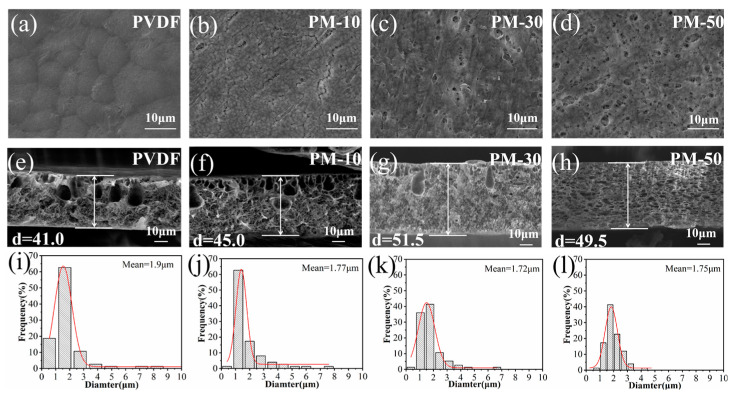
(**a**–**d**) SEM images of the top surface; (**e**–**h**) SEM images of a cross-section of PVDF, PM-10, PM-30, and PM-50; (**i**–**l**) cross-sectional pore size distribution of PVDF, PM-10, PM-30, and PM-50.

The microstructure of the MCH/PVDF composite membranes was analyzed using SEM. Figure 3 shows that all membranes have micropores on their top surfaces. The uniformity of these micropores was improved upon the addition of MCH nanofibers. Specifically, at a MCH nanofiber content of 50%, the pore size distribution of the composite membrane was the most uniform, with a size of 0.75 μm (Figure S1). Additionally, EDS mapping of fluorine (F) and magnesium (Mg) elements on the surface of PM-50 membranes indicated that MCH nanofibers were uniformly dispersed throughout the membrane (Figure S2).

The cross-sectional view in Figure 3 reveals a highly interconnected, porous structure in all membranes, characterized by finger-like pores on the top layer and dense micropores on the bottom layer. This asymmetrical morphology is a direct result of the phase inversion process, which greatly enhances the membrane’s porosity and ability to absorb liquid electrolytes.

The pore structure of the membranes created by the phase inversion method is significantly influenced by diffusion kinetics and thermodynamics during the membrane formation process [30]. The thermodynamic aspect relates to the phase equilibrium within a polymer solution component system, while the kinetic factor pertains to the mutual diffusion and convection of components [36]. Analysis of SEM cross-sections indicates that as the amount of MCH nanofibers increased in the composite membranes, finger-like pores decreased, while spongy micropores increased. Notably, at a MCH nanofiber content of 50%, through the composite membrane, spongy micropores were uniformly distributed. The pore size distributions at the cross sections of different membranes are shown in Figure 3i–l. Although the average pore size of PM-50 composite membrane is the same as that of PM-10, the pore size distribution analysis indicates that the range of pore size in the PM-50 composite membrane is much narrower than that in the PM-10 membrane. This is because as the MCHs nanofiber content increases, the amount of DMF solvent required in the mixture increases, resulting in a gradual decrease in the concentration of the casting solution, which gradually slows the polymer precipitation rate (i.e., the rate of conversion from solvent to non-solvent); the diffusion exchange between the solvent and non-solvent leads to the solution entering a thermodynamically unstable state, resulting in liquid phase separation [37]. According to Strathmann et al., a slow conversion rate leads to the formation of a spongy and uniform micropores structure [38]. A concentrated distribution of pore size would be beneficial to the formation of a uniform concentration gradient of lithium ions near the electrode side. 

### 3.3. Porosity and Electrolyte Uptake of Membranes

**Figure 4:**
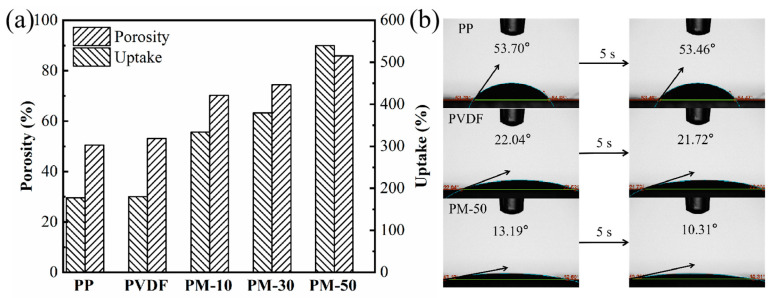
(**a**) Porosity and electrolyte uptake ratio; (**b**) contact angle testing of different membranes, showing the initial status (left) and latter status after 5 s (right).

For membranes to facilitate the efficient transport of lithium ions between the cathode and anode, they must exhibit excellent electrolyte wettability [39]. One significant consideration in achieving optimal liquid electrolyte absorption is the degree of porosity. Figure 4a illustrates a comparison of membrane porosity levels and corresponding electrolyte uptake. Composite membranes exhibit significantly higher porosity levels than PP (50.5%) and PVDF (53.1%). As the MCH nanofiber content increased in the composite membranes, their porosity levels also increased, with PM-50 (85.9%) exhibiting the highest porosity, followed by PM-30 (74.4%) and PM-10 (70.2%), respectively. Generally, higher porosity correlates with greater liquid electrolyte absorption, which is evident from the increasing trend of membrane electrolyte uptake observed in Figure 4a. The PM-50 membrane exhibits the highest electrolyte uptake ratio (539.8%) of all tested samples. This increase can be attributed to its spongy microporous structures formed by the addition of MCH nanofibers within the composite membrane, which promotes excellent compatibility with liquid electrolytes. We believe that the improved electrolyte affinity of the membranes is of great significance in enhancing the charging–discharging performance of LIBs.

The contact angle test results in Figure 4b were obtained to further evaluate the electrolyte absorption capabilities of various membranes. Due to its inherent low affinity with electrolytes, the PP membrane exhibited minimal absorption (contact angle: 53.7°), which remained relatively constant after 5 s. In contrast, the PM-50 membrane demonstrated exceptional electrolyte absorption abilities, with a contact angle of only 13.19°, and the electrolyte quickly infiltrated into the PM-50 membranes in 5 s. Spongy microporous structures within the membrane can enhance electrolyte uptake and solvated ion migration [40]. Additionally, strong interactions between the organic electrolyte and polar functional groups of PVDF also contribute to enhanced affinity between the membranes and electrolytes [41]. 

### 3.4. Thermal Stability and Flame-Retardant Capacity of Membranes

**Figure 5:**
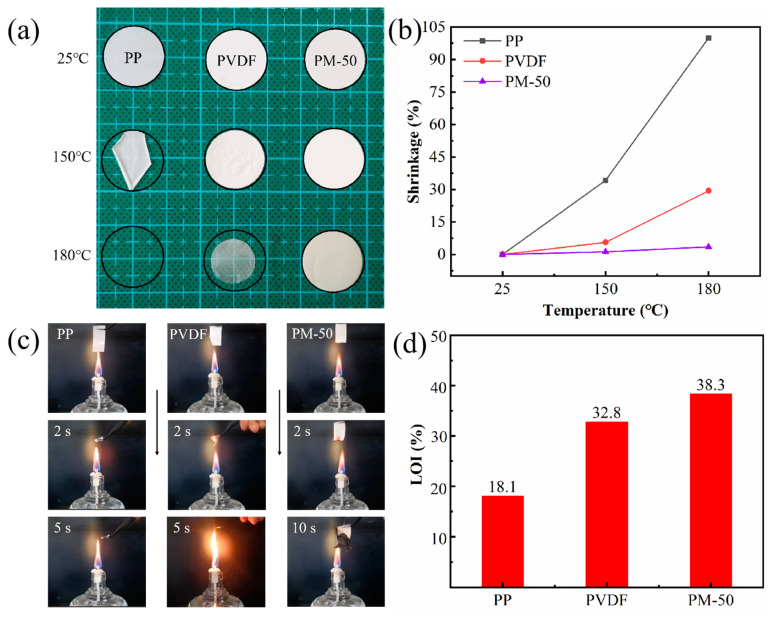
(**a**) Thermal shrinkage experiments at different temperatures; (**b**) thermal shrinkage ratios; (**c**) combustion experiments; (**d**) LOI values of different membranes.

The thermal stability of separators strongly impacts battery safety, as they must maintain their integrity and prevent physical contact between electrodes during temperature variations [42]. Traditional polyolefin membranes commonly exhibit severe shrinkage when subjected to high temperatures, thereby leading to significant safety concerns. It is generally recommended that membrane thermal shrinkage rates remain below 5% at higher temperatures to avoid short-circuiting within batteries [43]. Figure 5a shows the PP, PVDF, and PM-50 membranes after thermal treatments at different temperatures (25 °C, 150 °C, and 180 °C) for 2 h. Figure 5b displays the resulting thermal shrinkage ratios. Notably, the PM-50 membrane exhibits exceptional dimensional stability at up to 180 °C, with only minimal shrinkage, while PP and PVDF membranes experience irreversible structural damage; i.e., PP membrane shrinkage reached 100%, PVDF membrane shrinkage was 29.4%, and PM-50 membrane shrinkage only measured 3.5%.

In order to more directly prove the flame retardancy of composite membranes, Figure 5c shows camera images of PP, PVDF, and PM-50 membranes in burning tests. Apparently, when the PP membrane was close to the fire source, it shrank immediately and quickly burned within 5 s, and the PVDF membrane also shrank severely and became transparent liquid at 2 s. However, when the composite membrane was closed to the flame, the flame was extinguished immediately, and it still kept its original form quite well after 10 s. This phenomenon is mainly attributed to the fact that MCH nanofibers in composite membranes can decompose to generate water and CO_2_ (Figure S3), as shown in the following reaction formula:

At 100 °C:

$$4\mathrm{MgC}O_{3}\cdot \mathrm{Mg}{\left(\mathrm{OH}\right)}_{2}\cdot 5H_{2}O\to 4\mathrm{MgC}O_{3}\cdot \mathrm{Mg}{\left(\mathrm{OH}\right)}_{2}+5H_{2}O$$

At 350~400 °C:

$$4\mathrm{MgC}O_{3}\cdot \mathrm{Mg}{\left(\mathrm{OH}\right)}_{2}\to {\mathrm{Mg}}_{3}O{(CO_{3})}_{2}+2\mathrm{Mg}O+2CO_{2}↑+H_{2}O↑$$

At 400~600 °C:

$${\mathrm{Mg}}_{3}O{(CO_{3})}_{2}\to 3\mathrm{Mg}O+2CO_{2}↑$$

The MCH nanofibers release water vapor that dilutes the concentration of combustible gas within the flame area. This process facilitates the formation of a carbonized layer on the membrane surface, thereby preventing further oxidation. As MCH nanofibers decompose, they release CO_2_ gas that forms a dense, non-combustible layer of MgO on the membrane surface. This layer helps prevent combustion while also removing heat through an endothermic process, reducing the risk of flame propagation and achieving effective flame retardancy.

To evaluate the flame-retardant capabilities of the membranes, we also conducted limiting oxygen index (LOI) tests, and present the results in Figure 5d. The LOI value represents the minimum oxygen volume percentage that can support material combustion, and serves as an indicator of flammability. Combustible materials typically have LOI values of 22–27%, with higher values indicating lower combustibility. As expected, the LOI value of the PP membrane is 18.1%, which indicates that PP is a flammable material. In contrast, the PM-50 membrane could be certificated as a flame-retardant material with a high LOI value of 38.3% (GB/T 2406-93 [44]). Such enhanced flame-retardant capabilities in composite membranes will undoubtedly improve the safety of LIBs.

### 3.5. Electrochemical Properties 

**Figure 6:**
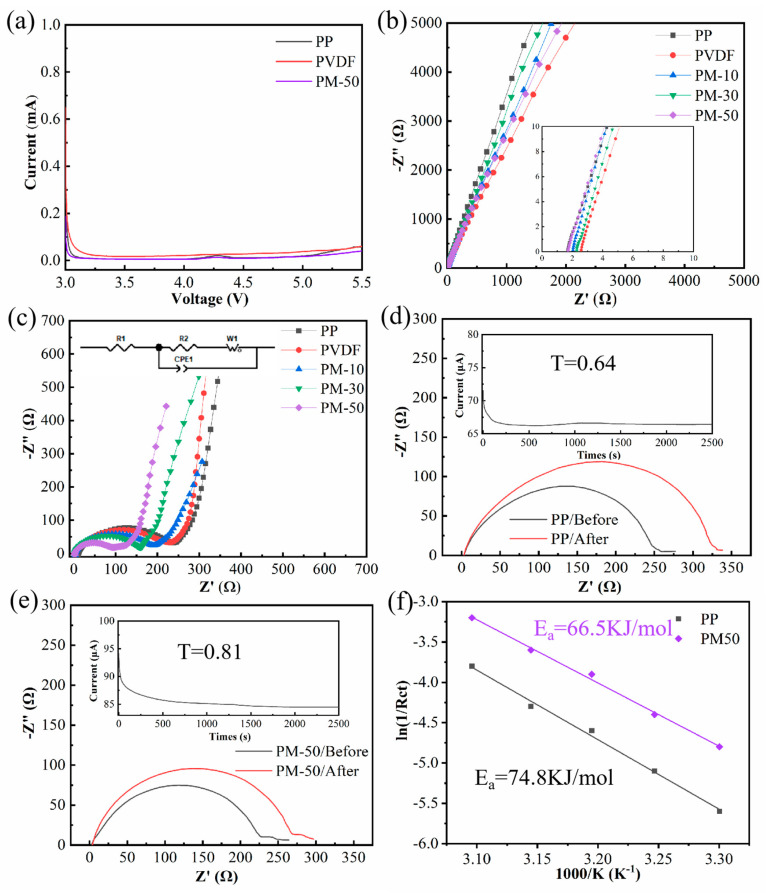
(**a**) LSV curves of cells with different membranes; (**b**) Nyquist plots of SS/membrane/SS cells; (**c**) Nyquist plots of LiFePO_4_/membrane/Li cells (inset: equivalent circuit for fitting impedance spectra); (**d**) lithium ion transference number of symmetric Li/PP/Li cells; (**e**) lithium ion transference number of symmetric Li/PM−50/Li cells; (**f**) different activation energies for lithium ion transport.

Safety concerns can arise in LIBs due to the decomposition of oxidative electrolytes on electrodes, which can be evaluated by measuring the electrical current when increasing the voltage, in order to determine the voltage limit for the cell [10]. An LSV analysis was therefore used to assess the electrochemical stability of cells with PP, PVDF, and PM-50 membranes. Figure 6a demonstrates that cells with a composite membrane exhibit a similar voltage window to cells with a commercial PP membrane. This indicates that cells with a composite membrane can stably operate within the charge–discharge voltage range (i.e., 3–5.5 V).

Ionic conductivity is a crucial parameter to evaluate membrane performance in LIBs; it was measured via EIS (Figure 6b) and calculated using Equation (4). R_b_ is the intercept on the X axis of a Nyquist plot in a high-frequency range. Cells with MCH/PVDF composite membranes showed higher ionic conductivity when compared to cells with PP and PVDF membranes, especially, displaying an incremental trend as MCH nanofiber content gradually increased (Table 1). This increase can be attributed to the higher porosity and electrolyte uptake of composite membranes.

To examine the compatibility between a membrane impregnated with liquid electrolytes and electrode materials, AC impedance spectra were used to evaluate the interfacial impedance of LiFePO_4_/membrane/Li cells assembled with different membranes. The Nyquist plots in Figure 6c show semicircles in the mid-frequency range, representing the charge transfer resistance (R_ct_) in the interfacial region [45]. The LiFePO_4_/PM-50/Li cells demonstrate the lowest R_ct_ (93.3 Ω) due to their higher porosity and superior electrolyte wettability. The lower R_ct_ can facilitate the efficient transport of lithium ions between the electrode and electrolyte, ultimately improving the charging and discharging performance of the cell.

For LIB systems, increasing the lithium ion transference number (t_Li_^+^) can minimize anion movement and decrease the polarization effect during battery operation [46,47]. This is significant for achieving high coulombic efficiency and delaying the formation and growth of lithium dendrites on the cathode’s surface. We evaluated the t_Li_^+^ of PP and PM-50 membranes using chronoamperometry and AC impedance spectroscopy techniques, and the results are shown in Figure 6d,e. The calculated results show that symmetric Li/Li cells with PM-50 membranes have a higher t_Li_^+^ value of 0.81 compared to cells with PP membranes (t_Li_^+^ = 0.64), indicating that incorporating MCH nanofibers can considerably enhance lithium ion migration efficiency within electrolyte-absorbing membranes; the specific relevant values for t_Li_^+^ calculation are shown in Table S1. The results demonstrated the spongy microporous structure of the MCH/PVDF composite membranes greatly improves the migration efficiency of lithium ions. Besides, the hydroxyl functional group on the surface of MCH nanofibers and the lithium salt anion (PF_6_^−^) in the electrolyte can form a strong binding force through hydrogen bonding, thereby limiting the transfer of anions. Charge transfer resistance (R_ct_) at different temperatures was investigated to evaluate the energy barrier during lithium ion transport at the interfaces of electrode and electrolyte-absorbing membrane (Figure S4). The activation energy for lithium ion transport can be obtained from a linear fit of the Arrhenius equation (Equation (7)). The symmetric Li cell with a PM-50 composite membrane shows an activation energy of 66.5 kJ/mol, lower than that of the cell with a PP membrane (74.8 kJ/mol) (Figure 6f), demonstrating that solvated lithium ions can be desolvated and migrate faster at the interfaces of electrodes and electrolyte-absorbing membranes.

**Figure 7:**
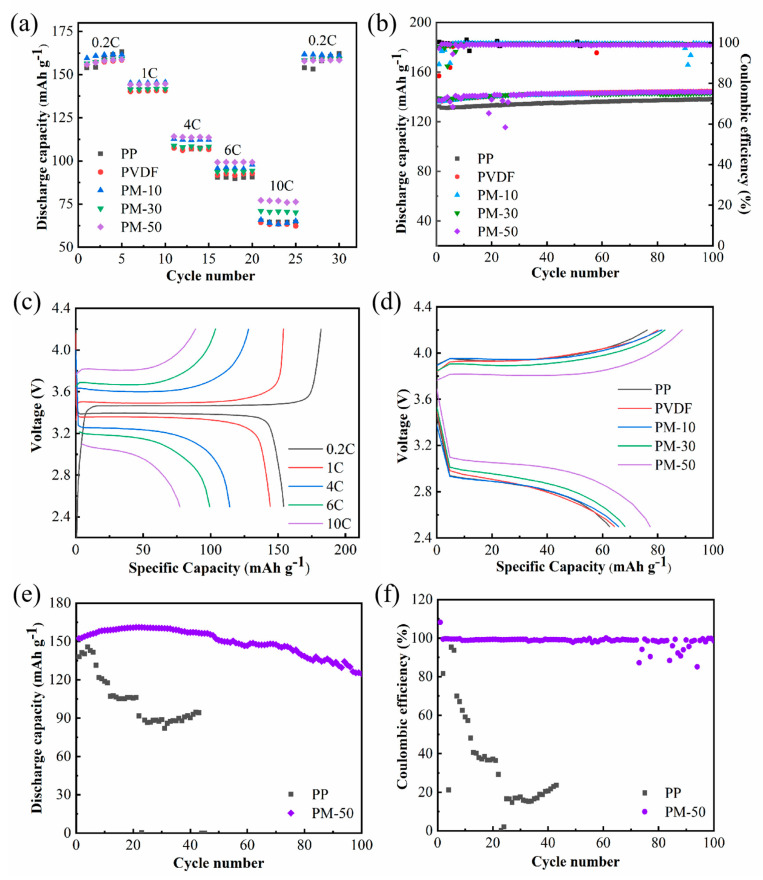
(**a**) C−rate performance of cells; (**b**) cycling tests of cells at 1 C; (**c**) the charge−discharge profiles of cells with PM−50 at various rates; (**d**) the charge−discharge profiles of cells with different membranes at 10 C; (**e**) discharge capacity and (**f**) coulombic efficiency of cells with different membranes at 100 °C.

We assembled LiFePO_4_/membrane/Li half-cells to evaluate their electrochemical performance with different membranes. Rate capability tests from 0.2 to 10 C (and back down to 0.2 C) were conducted, and the results are shown in Figure 7a. The PM-50 cell yielded specific discharge capacities of 152.6, 144.2, 114.2, 99.3, and 77.2 mAh·g^−1^ at 0.2, 1, 4, 6, and 10 C, respectively. Additionally, when the charging/discharging rate returned to 0.2 C, the discharge capacity could almost be recovered (151.8 mAh·g^−1^). This excellent rate capability indicates resilience during charging/discharging cycles. As the C-rate increased, a much higher discharge capacity was observed in the PM-50 cell compared to cells with PP and PVDF membranes.

We conducted cycling tests of LiFePO_4_/membrane/Li half-cells at 1C (Figure 7b). The LiFePO_4_/PM-50/Li cell exhibited an excellent initial discharge capacity of 137.3 mAh·g^−1^, which only slightly increased to 144.0 mAh·g^−1^ after 100 cycles, achieving over 99% coulombic efficiency (Figure 7b). A stable cycling performance was observed in a cell with a PM-50 membrane.

Figure 7c shows the charge–discharge voltage profiles of cells assembled with PM-50 at various rates during the first cycle. The potential plateau (~3.4 V) can be attributed to the Fe^2+^/Fe^3+^ redox reaction [48,49]. As indicated in Figure 7d, compared to other membranes, the LiFePO_4_/PM-50/Li cell exhibited higher capacity, even at 10 C, due to its superior ionic conductivity. The cell with PM-50 shows minimal voltage polarization compared to other cells.

We also performed a cycling test at 100 °C to compare the cycling stability of cells with different membranes. Figure 7e,f show the discharge capacities and coulombic efficiencies of the cells as a function of cycle number. LiFePO_4_/PP/Li half cells experienced significant capacity fading, unlike cells assembled with PM-50 that exhibited superior capacity retention and coulombic efficiency. The capacity increased during the first twenty cycles due to cell activation. Our study provides an opportunity to operate LIBs at high temperatures, combined with a high-rate performance for future practical use.

**Figure 8:**
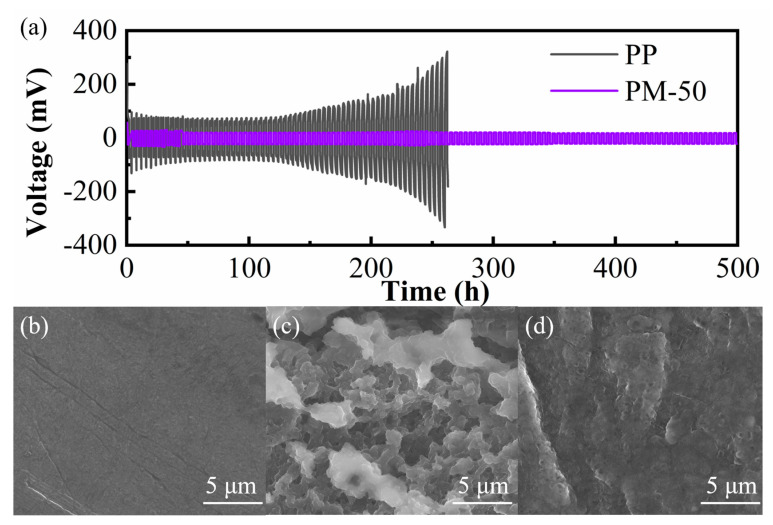
(**a**) Galvanostatic cycling profiles of symmetric Li/membrane/Li cells with PP and PM−50 at a current density of 0.5 mA cm^−2^; SEM images of a Li electrode (**b**) before cycling, and after cycling for 500 h with (**c**) PP and (**d**) PM−50.

The Li/membrane/Li cells were assembled with PP and PM-50, respectively, and subjected to cycling tests at a current density of 0.5 mA/cm^2^; therefore, lithium ions were repeatedly plated and stripped in order to characterize the compatibility of the membranes with the electrodes. As shown in Figure 8a, the Li/PP/Li cell shows an obvious voltage fluctuation at 140 h, with the polarized voltage rapidly increasing from 90 mV to 300 mV, whereas the voltage of the Li/PM-50/Li cell remains stable at 500 h, indicating that the composite membrane has excellent interfacial compatibility with the lithium electrode surfaces. This can mainly be attributed to the homogeneous spongy microporous structure of the PM-50 composite membrane. Meanwhile, SEM was used to observe the microscopic morphology of the lithium surface before and after cycling. As shown in Figure 8b, the lithium metal had a smooth surface before cycling. A lot of dendritic and mossy lithium dendrites were generated on the electrode surface in the symmetric cell with a PP membrane after cycling (Figure 8c). In contrast, the electrode surface in the cell with PM-50 was still relatively smooth (Figure 8d); there was no obvious generation of dendrites. In conclusion, the PM-50 composite membrane was able to achieve a uniform concentration gradient and deposition of lithium ions, thus reducing the short-circuiting risks caused by lithium dendrites’ growth.

## 4. Conclusions

We successfully prepared MCH/PVDF composite membranes using the NIPS method, and investigated their possible applications as separators for LIBs. The addition of MCH nanofibers into PVDF had a positive impact on the porous morphology, electrolyte affinity and electrochemical properties of composite membrane. As the content of MCH nanofibers increased in the polymer matrix, PM-50 composite membranes displayed higher electrolyte uptake (539.8%), ionic conductivity (1.4 mS·cm^−1^), a higher lithium ion transference number (0.81), and a lower activation energy for desolvation (66.5 kJ/mol) and interfacial resistance (93.3 Ω). An excellent rate (77.2 mAh·g^−1^ at 10 C) and cycling performance (99% capacity retention after 100 cycles at 1 C) were observed in the LiFePO_4_/PM-50/Li half cells. Cells assembled with PM-50 maintained superior charging–discharging performance after 100 cycles at 100 °C, with ~100% coulomb efficiency. Furthermore, the MCH/PVDF composite membranes exhibited excellent thermal stability and flame retardancy. These findings suggest that MCH/PVDF composite membranes are a potential separator candidate for future high-rate and high-safety advanced LIBs, due to their superior overall properties. Overall, our study sheds light on the future development of high-rate and safe LIBs.

## Figures and Tables

**Table 1:** Resistance (R_b_ and R_ct_) and ionic conductivity (σ) of symmetrical SS/membrane/SS cells with different membranes.

Sample	D (μm)	R_b_ (Ω)	σ (mS cm^−1^)	R_ct_ (Ω)
PP	25.0	1.7	0.7	204.7
PVDF	41.0	2.6	0.8	195.5
PM-10	45.0	2.0	1.1	190.8
PM-30	51.5	2.3	1.2	159.1
PM-50	49.5	1.7	1.4	93.3

## Data Availability

Data is available upon request.

## Supplementary Materials

The following supporting information can be downloaded at: https://www.mdpi.com/article/10.3390/polym15204120/s1, Figure S1: Pore size distributions of (a) PM−10, (b) PM−30, and (c) PM−50 membranes; Figure S2: EDS mapping of PM−50 membranes with F and Mg elements; Figure S3: TG curve of MCH nanofibers; Figure S4: The charge transfer resistances (R_ct_) at different temperatures of symmetric Li/Li cells with (a) PP and (b) PM−50 membranes; Table S1: The specific values for lithium ion transference number calculation.
