# Supplementary material for: A Magnesium Carbonate Hydroxide Nanofiber/Poly(Vinylidene Fluoride) Composite Membrane for High-Rate and High-Safety Lithium-Ion Batteries
Source: Polymers (Basel). 2023 Oct 17;15(20):4120. doi: 10.3390/polym15204120 (PMC10611082; doi:10.3390/polym15204120)
Supplement: Supplementary file 1 [file polymers-15-04120-s001.zip › polymers-2646803-supplementary.pdf]

Supporting information

Supplementary Materials

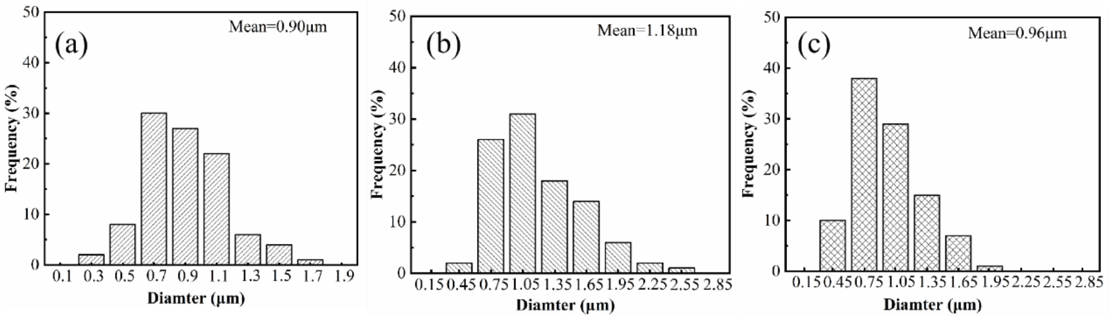

Figure S1. Pore size distributions of (a) PM-10, (b) PM-30, and (c) PM-50 membranes.

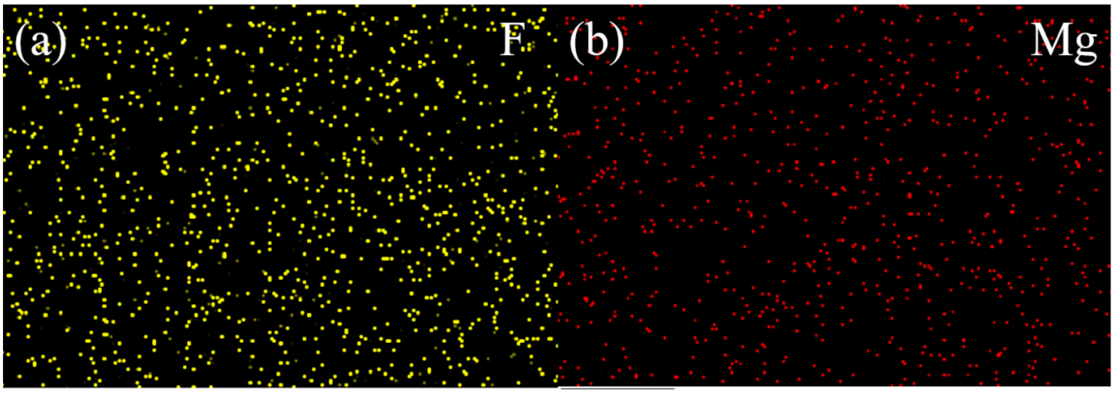

Figure S2. EDS mapping of PM-50 membranes with F and Mg elements.

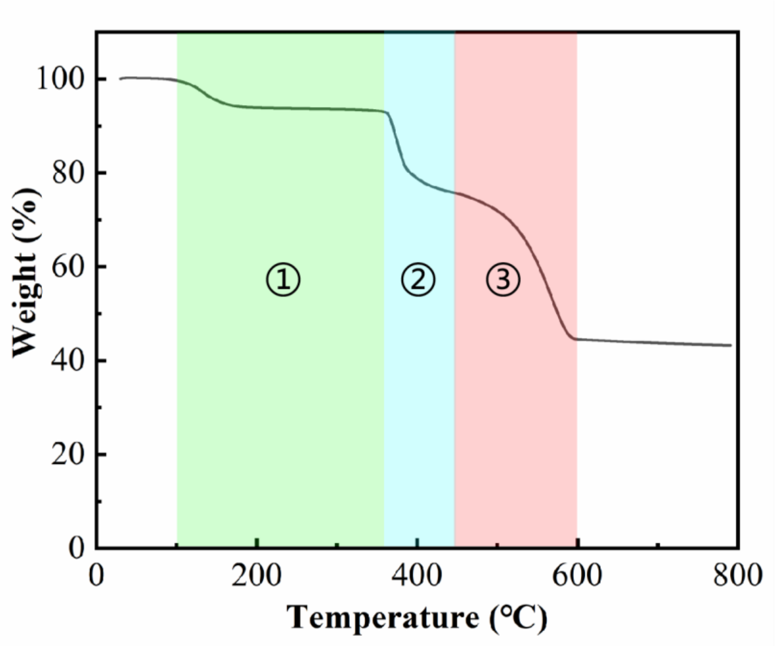

Figure S3. TG curve of MCH nanofibers.

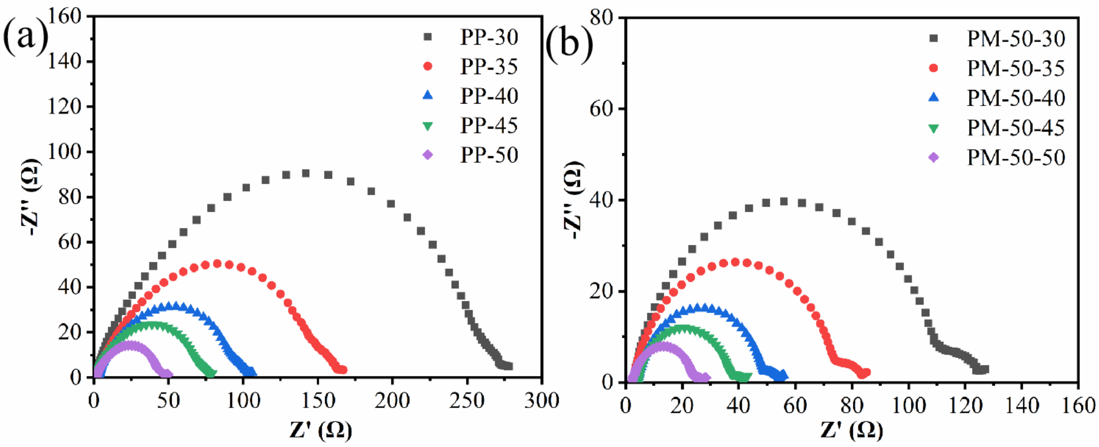

Figure S4. The charge transfer resistances (R_{ct}) at different temperatures of symmetric Li/Li cells with (a) PP and (b) PM-50 membranes.

Table S1. The specific values for lithium ion transference number calculation

cell	I_0 (μA)	I_S (μA)	R_0 (Ω)	R_S (Ω)	ΔV (V)	t_{Li^+}
**Li/PP/Li**	70.7	66.4	255.6	328.7	0.01	0.64
**Li/PM-50/Li**	94.2	84.5	229.9	270.5	0.01	0.81
